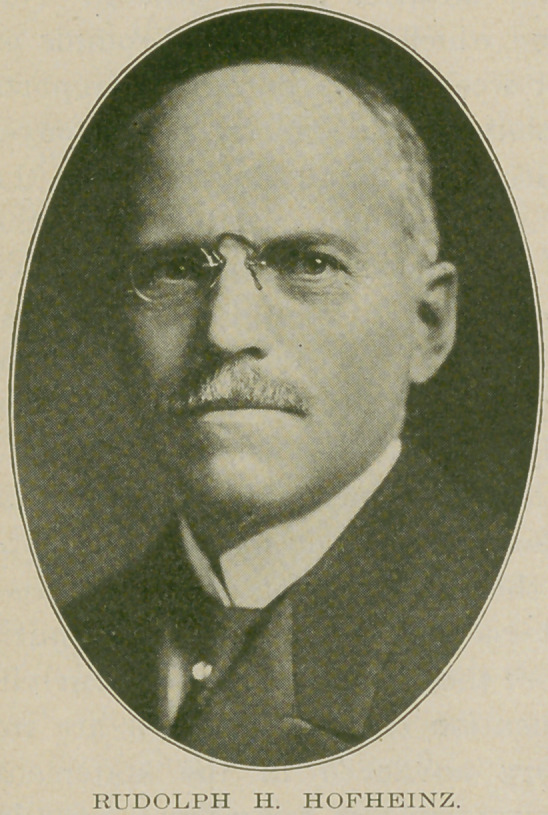# Obituary

**Published:** 1917-01

**Authors:** 


### OBITUARY

RUDOLPH H. HOFHEINZ.

HOFHEINZ, RUDOLPH H. Born in 1856. Died November 23, 1916. Dr. Hofheinz was born in Heidelberg, Germany and spent his youth there, coming to America after the death of his parents in 1871. He was graduated from the Now York College of Dentistry in 1879 with the highest honors of his class, after which he came to Rochester where he has practiced until the time of his death. From 1896 to 1909 Dr. Hofheinz was professor of operative dentistry in the University of Buffalo, and upon his retirement was appointed Professor Emeritus. He was from the first active in dental society affairs and always took a prominent part in various dental activities. Dr. Hofheinz was president of the Seventh District, the New York State Dental Society and the Rochester Dental Society, and also a member of the Committee of Organization of the Fourth International Dental Congress, filling the various positions with signal honor to himself and to the great satisfaction of those whom he served. He took an active part in the organization of the Rochester Dental Dispensary and at the time of his death was a director and vice-president. On October 24th, of this year, he was appointed principal of the School for Dental Hygienists of the Rochester Dental Dispensary, to which he had expected to devote much of his time, energy and ability. Dr. Hofheinz was always a student, and one of the most highly educated men in dentistry. He was a frequent contributor to the literature of the profession, an essayist of distinction and a discussor of papers of pleasing and rare ability. Few men have occupied so large a place in dental affairs as did he. He enjoyed a wide acquaintance among the older men in dentistry. To the younger men and those just entering the profession, he was most considerate and helpful, and many are there who owe much of their success to his kindly advice and assistance. He had a kindly and genial disposition, and was the friend of everyone. He was a true and devoted friend, and was loved most where he was known best. His death is a distinct loss not only to the profession which he served so long and devotedly, but to a wide circle of friends who loved him for his many fine qualities of mind and heart. Dr. Hofheinz is survived only by his devoted wife, to whom he was married in 1884.—H. J. B.

## Figures and Tables

**Figure f1:**